# Presynaptic congenital myasthenic syndrome with altered synaptic vesicle homeostasis linked to compound heterozygous sequence variants in *RPH3A*

**DOI:** 10.1002/mgg3.370

**Published:** 2018-02-14

**Authors:** Ricardo A. Maselli, Jessica Vázquez, Leah Schrumpf, Juan Arredondo, Marian Lara, Jonathan B. Strober, Peter Pytel, Robert L. Wollmann, Michael Ferns

**Affiliations:** ^1^ Department of Neurology University of California Davis Sacramento CA USA; ^2^ Department of Neurology University of California San Francisco San Francisco CA USA; ^3^ Department of Pathology University of Chicago Chicago IL USA; ^4^ Department of Anesthesiology and Pain Medicine University of California Davis Sacramento CA USA

**Keywords:** congenital myasthenic syndrome (CMS), presynaptic, rabphilin 3a, *RPH3A*

## Abstract

**Background:**

Monogenic defects of synaptic vesicle (SV) homeostasis have been implicated in many neurologic diseases, including autism, epilepsy, and movement disorders. In addition, abnormal vesicle exocytosis has been associated with several endocrine dysfunctions.

**Methods:**

We report an 11 year old girl with learning disabilities, tremors, ataxia, transient hyperglycemia, and muscle fatigability responsive to albuterol sulfate. Failure of neuromuscular transmission was confirmed by single fiber electromyography. Electron microscopy of motor nerve terminals revealed marked reduction in SV density, double‐membrane‐bound sacs containing SVs, abundant endosomes, and degenerative lamellar bodies. The patient underwent whole exome sequencing (WES) and relevant sequence variants were expressed and studied in a mammalian cell line.

**Results:**

Chromosomal microarray studies and next generation sequencing (NGS) of mitochondrial DNA were unrevealing; however, NGS of genomic DNA showed two rare sequence variants in the gene encoding rabphilin 3a *(RPH3A)*. The paternally inherited variant c.806 G>A (p.Arg269Gln) involves a substitution of a conserved residue in the linker region, while the maternally inherited variant c.1390 G>T (p.Val464Leu) involves a conserved amino acid substitution in the highly conserved C2A region. Expression studies revealed that p.Arg269Gln strongly impairs the binding of rabphilin 3a to 14‐3‐3, which is a proposed regulator of synaptic transmission and plasticity. In contrast, the binding of rabphilin 3a to 14‐3‐3 is only marginally impaired by p.Val464Leu; thus, the pathogenic role of p.Val464Leu remains unclear.

**Conclusion:**

In summary, we report a patient with a multisystem neurologic disorder and altered SV regulation attributed to defects in *RPH3A*, which grants further studies of this gene in human disorders of synaptic transmission.

## 1 INTRODUCTION

Congenital myasthenic syndromes (CMS) are a diverse group of neurologic diseases of neuromuscular transmission that are characterized by muscle weakness and fatigability (Engel, Shen, Selcen, & Sine, 2015). The majority of CMS result from genes defects in proteins of the postsynaptic compartment. However, in recent years the introduction of whole exome sequencing (WES) into clinical practice has allowed the discovery of many rare forms of CMS due to defects in genes encoding proteins of the presynaptic apparatus. Most of these newly described presynaptic CMS are caused by defects in proteins associated with the SNARE (soluble *N*‐ethylmaleimide‐sensitive factor attachment protein receptor) complex, which is the core process behind synaptic vesicle (SV) fusion and exocytosis (Engel, Selcen, Shen, Milone, & Harper, 2016; Shen, Selcen, Brengman, & Engel, 2014; Shen et al., 2017).

**Figure 1:**
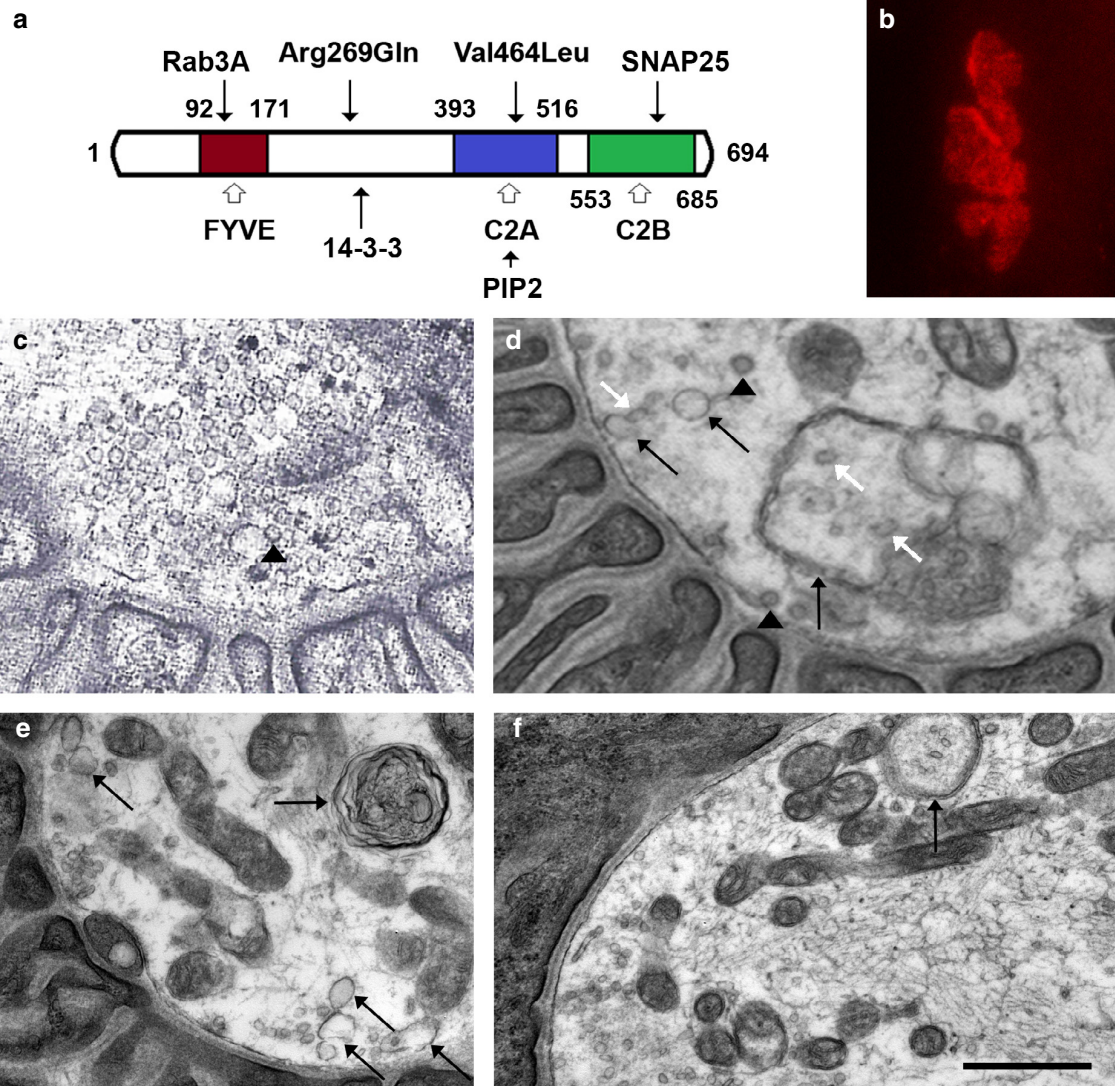
Rabphilin 3a domains and pathological findings: a. Schematic representation of rabphilin 3a showing the locations of the variants and the most important sites of interactions with other proteins. The FYVE (or RBD) domain encompasses the Zn binding site for Rab3a and the Arg269Gln variant lies adjacent to the binding site for 14‐3‐3. The Val464Leu variant is located in the C2A domain and the C2B domain mediates binding to SNAP25. b. Endplate of the patient stained with rhodamine‐αBGT demonstrating normal size and expression of AChRs. c**.** Normal NMJ of a control patient. d. A NMJ of the patient showing severe depletion and variable shapes of SVs (black arrowheads point to a coated SVs and oblique white arrows point to elliptic shaped SVs), abundant endosomes (oblique black arrows) and a DMBS containing SVs (vertical black arrow). e. A NMJ showing a degenerative lamellar body (horizontal black arrow) and abundant endosomes. f. A NMJ showing a DMBS containing SVs. The calibration mark represents 14 μm in B, 0.5 μm in c and d, and 0.8 μm in e and f

Rabphilin 3a is an evolutionary conserved molecule that interacts with the small GTP‐binding proteins Rab3A and Rab27a, and it participates in the trafficking and release of both synaptic and secretory vesicles (Shirataki et al., 1993). Rabphilin 3a consists of an N‐terminal domain that binds Rab3a and Rab27a, a linker region that binds 14‐3‐3, and two tandem C2 domains that bind Ca^2+^ and phosphatidylinositol 4,5‐bisphosphate (PIP2) (Kato et al., 1996; Shirataki et al., 1993; Sun, Bittner, & Holz, 2003) (Figure 1a). The second C2 domain (C2B) also mediates specific interactions with SNAP25 and CASK (Tsuboi & Fukuda, 2005). Moreover, similar C2 domains are found in several other molecules that are also involved in SV trafficking, docking and fusion, including synaptotagmin, Doc2, Munc13 and RIM (Corbalan‐Garcia & Gómez‐Fernández, 2014).

The precise role of rabphilin 3a in synaptic transmission is complex and poorly understood. Deletion of the rabphilin 3a gene (*RPH3A)* (OMIM 612159) in mice and *Caenorhabditis elegans* resulted in no obvious phenotypic effects (Schlüter et al., 1999; Staunton, Ganetzky, & Nonet, 2001), but facilitated recovery of responses at depressed synapses (Deák et al., 2006). Furthermore, microinjection of exogenous rabphilin 3a inhibited SV exocytosis in squid giant axons (Burns, Sasaki, Takai, & Augustine, 1998). Together, this suggests that rabphilin 3a may act as a brake on SV release in neurons. In contrast, overexpressing rabphilin 3a enhanced hormonal release in bovine chromaffin and PC12 cells, suggesting that it promotes exocytosis in neuroendocrine cells (Chung, Takai, & Holz, 1995). Thus, it seems that under different conditions rabphilin 3a can exert either positive or negative effects on exocytosis and downstream endocytosis (Deák et al., 2006).

We report here an individual with a mild form of CMS and additional neurologic manifestations attributed to mutations in the rabphilin *RPH3A*, in whom an electron microscopy (EM) study of motor nerve terminals revealed signs consistent with altered exocytosis and downstream endocytosis.

## 2 CLINICAL REPORT

The patient is an 11 year old girl, who was born full‐term to a nonconsanguineous couple and had an uncomplicated perinatal period. All early motor milestones were achieved on target, but during early childhood her parents noticed limb weakness and poor physical endurance. At age 3 she developed hand tremors, incoordination in hands and postural imbalance (Video S1). At age 5 she developed hyper‐nasal speech and a flexible laryngoscopy confirmed the presence of velopharyngeal insufficiency. She also has history of recurrent abdominal pains and transient hyperglycemia. She had learning disabilities, particularly for word reading, reading comprehension and spelling, but brain MRI and EEG were normal. Repetitive stimulation of the left spinal accessory and ulnar nerves revealed no decrement of compound muscle action potential (CMAP) amplitudes with stimulation at 2 Hz, but an increment of 30% of CMAP areas was noted with stimulation at 30 Hz. Serum antibodies against AChR and MuSK were negative. A biopsy of the right quadriceps muscle at age 6 and the left anconeus muscle at age 8 revealed no significant abnormalities at the light microscopy level. Single fiber EMG by axonal stimulation in the right deltoid muscle performed at the time of her anconeus biopsy revealed increased jitter consistent with failure of neuromuscular transmission. She had obstructive sleep apnea, which partially subsided after tonsillectomy. A recent neurologic examination revealed no dysmorphic features, and normal behavior for age. She had no ptosis and normal external ocular movements. She had mild weakness of neck flexors and proximal muscles of the upper extremities with normal deep tendon reflexes. Coordination testing revealed moderate dyssynergia to finger‐to‐nose test and impaired rapid alternating movements of hands. She had difficulties to perform tandem gait.

To investigate the nature of the neuromuscular deficit part of the material from the anconeus muscle biopsy was used for structural endplate studies, which included acetylcholinesterase (AChE) reaction in teased muscle fibers, alpha‐bungarotoxin (αBGT) staining of endplates and EM of the neuromuscular junction (NMJ). Light microscopy showed only type I predominance; however, low magnification EM revealed occasional myofibers with nonspecific filament bodies and mild Z‐disk disruption. The staining with AChE and αBGT showed endplates of normal size and distribution with normal expression of AChE and AChR (Figure S1 and Figure 1b).

The EM studies of the NMJs identified three distinct changes: First, there was a reduction in the density of SVs, but the average diameter of SVs in the patient's muscle was not different from controls (Figure 1c–d). Second, there was an increase in nonvesicular synaptic membranes, including abundant endosomes, coated pits and double‐membrane bound sacs (DMBS) containing SVs (Figure 1d–f). Third, there were degenerative lamellar bodies (Figure 1e).

The morphometric analysis revealed that in comparison to control anconeus muscles, the patient showed no reduction in postsynaptic folding expressed as the endplate index (ratio of postsynaptic membrane length/presynaptic membrane length) (Table S1). The average width of the primary synaptic cleft and the average axon terminal area were also normal.

## 3 MATERIALS AND METHODS

To elucidate the genetic bases of the proband's disease she had a karyotype study, which showed a balanced translocation of part of the long arm of the X chromosome onto the short arm of chromosome 11 [46,X, t(X;11)(q26.1;p15.3)], but there was no significant rearrangements of nuclear or mitochondrial DNA detected by a comparative genomic hybridization microarray. Furthermore, no large deletions or deleterious point mutations of mitochondrial DNA were identified by next generation sequencing. Next, whole exome sequencing (WES) was performed as described in the Supporting Information Method Section and the variants of interest were confirmed by Sanger sequencing. The study was approved by the institutional review board of the University of California Davis and all participating individuals signed a consent form.

## 4 RESULTS

Analysis of WES revealed single nucleotide variants in three genes with potential association with the phenotype of the patient (Tables S2 and S3). The genes were *ALG13* (OMIM 300776), *DOK7* (OMIM 610285), and *RPH3A*. *ALG13* along with *ALG14* (OMIM 612866) encodes the UDP‐GlcNAc transferase, which catalyzes a key step in endoplasmic reticulum N‐linked glycosylation Since mutations in *ALG14* have recently been reported in association with CMS (Cossins et al., 2013) any rare sequence variant in *ALG13* was considered potentially relevant. The patient was a carrier of a heterozygous c.1154 A>G change resulting in p.Glu385Gly in *ALG13*, which is a rare variant [G = 0.0003/1 (1000 Genomes)]. However, the effect of this variant that the patient inherited from her unaffected mother is predicted to be tolerated by the SIFT and PolyPhen‐2 programs. Furthermore, the phenotype associated with mutations in ALG13 is one of epileptic encephalopathy not present in either the patient or the mother, who both carry the same sequence variation. Thus, Glu385Gly in *ALS13* was considered unlikely to be causative.

Both the patient and her unaffected mother were found to be heterozygous for the *DOK7* pathogenic mutation 1124_1127dupTGCC. However, Sanger sequencing failed to detect a second mutation in the same gene. Nevertheless, since intragenic microdeletions in *DOK7* are possible*,* an analysis of coding and untranslated regions of this gene was performed using a high density *DOK7‐*specific array, which was also normal (Figure S2). Thus, since *DOK7* myasthenia is recessive, the heterozygous mutation found in this gene was also considered unlikely to be causative.

By contrast, given the implications of rabphilin 3a in SV vesicle trafficking and neurotransmitter release, mutations in *RPH3A* were considered relevant to the phenotype of the patient.

**Figure 2:**
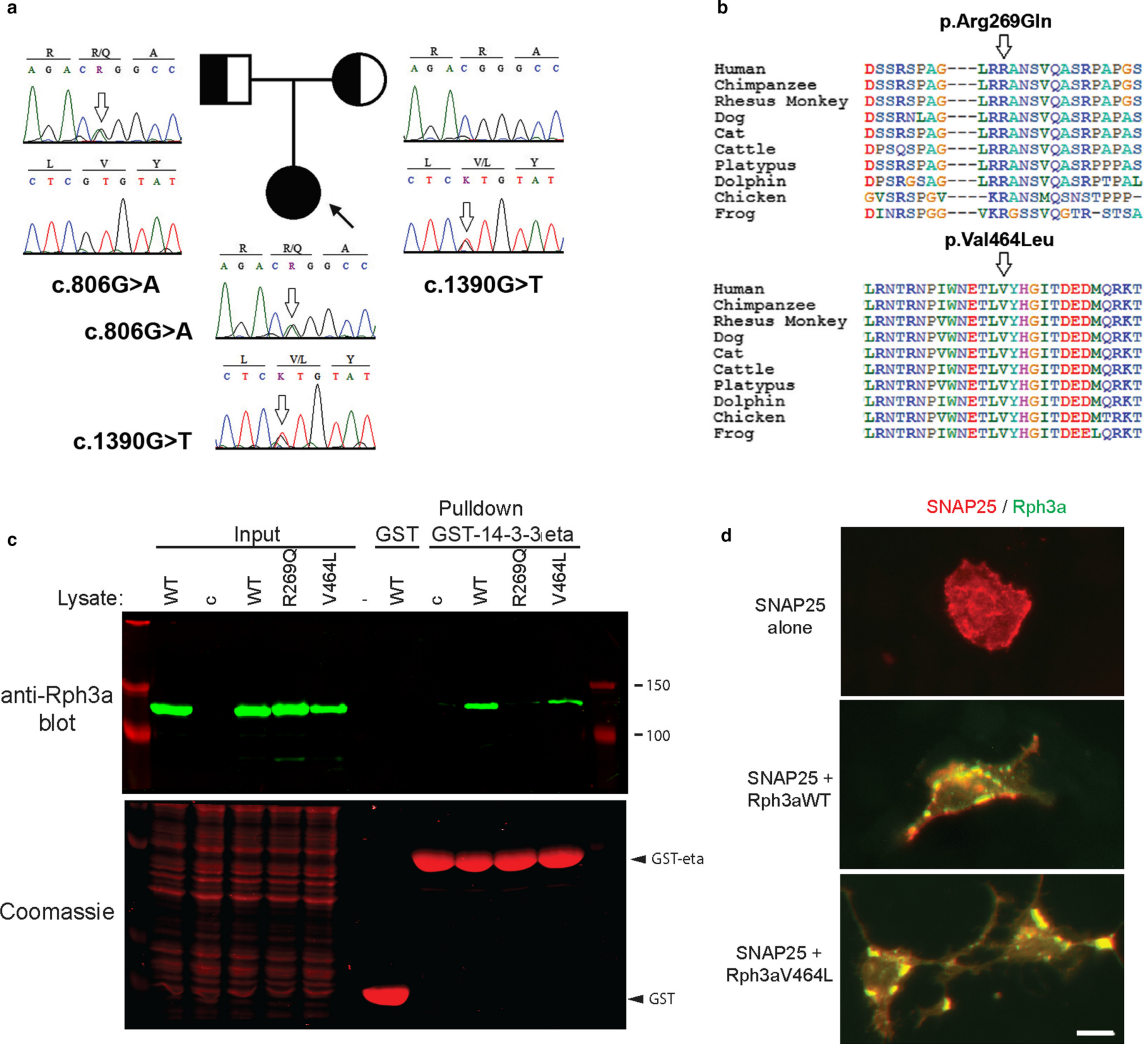
Pedigree, alignment of variants and expression studies. a. Family tree showing the segregation of *RPH3A* variants. b. Sequence alignments across species showing the location of the variants at conserved domains of the protein. c. Assay of rabphilin 3a–14‐3‐3 binding. Rabphilin 3a (Rph3a) variants were expressed in HEK cells and the cell lysates incubated with GST or GST‐14‐3‐3 eta beads. Rabphilin pulled down on the beads was detected by immunoblotting with anti‐rabphilin 3a antibody (upper panel) and GST‐fusion proteins were detected by Coomassie staining (lower panel). Control (c) is the cell lysates of sham‐transfected cells. Rabphilin 3a pulldown by GST‐14‐3‐3 eta was severely reduced by the Arg269Gln mutant, but only marginally by Val464Leu compared to rabphilin 3a WT. d. Rabphilin 3a colocalization with SNAP25. HEK cells transfected with rabphilin 3a‐YFP, Rab3a and/or SNAP25 were immunostained with SNAP25 antibodies. When expressed alone, SNAP25 was diffusely distributed, but when co‐expressed it partially colocalized with rabphilin in vesicular bodies (yellow puncta) near the cell membrane. Both rabphilin 3a WT and Val464Leu induced a similar redistribution of SNAP25. The calibration mark represents 10 µm

The two variants identified in *RPH3A* by WES were validated by Sanger sequencing and confirmed that each one derived from each parent (Figure 2a). The paternally inherited variant c.806 G>A, p.Arg269Gln (rs373497170) is rare [A = 0.0008/50 (ExAC)], involves a nonconservative amino acid substitution of a residue that is conserved among most species (Figure 2b) and is predicted to be pathogenic by SIFT and PolyPhen‐2 HumVar. The p.Arg269Gln change is located in the linker region following the Rab3A binding site (residues 70–140) and neighbors Ser‐272, which is the likely phosphorylated residue to which 14‐3‐3 binds (Sun et al., 2003).

The maternally inherited variant c.1390 G>T, p.Val464Leu (rs35555961) is rare [T = 0.0007/84 (ExAC)] and involves a conservative amino acid substitution of a residue that is conserved across most species (Figure 2b). While p.Val464Leu is predicted to be tolerated by SIFT and PolyPhen‐2HumVar it lays in the highly conserved fifth beta strand of the C2A domain, which mediates binding of Ca^2+^ and PIP2 (Coudevylle, Montaville, Leonov, Zweckstetter, & Becker, 2008).

To investigate the impact of p.Arg269Gln and p.Val464Leu we introduced them in a human *RPH3A* clone and studied the effect of the variants on binding to known partners, including 14‐3‐3, Rab3a and SNAP25 (Supporting Information Method Section). To assay 14‐3‐3 interaction, we expressed the rabphilin 3a constructs in HEK cells by transient transfection, and then the solubilized cell lysates were incubated ~ 1 hr with GST‐14‐3‐3 eta fusion protein purified on Glutathione 4B beads. After washing, rabphilin 3a protein bound to the GST‐14‐3‐3 beads was eluted and detected by immunoblotting with anti‐rabphilin 3a antibody. All rabphilin 3a variants were expressed at similar levels in the cell lysates and, as expected, we detected robust pulldown of rabphilin 3a WT by GST‐14‐3‐3 eta but not by GST control beads (Figure 2c). Notably, pulldown of rabphilin 3a p.Arg269Gln was severely reduced compared to wild type (8 ± 3% of WT levels; n = 4, p < 0.001, Student's t‐test), whereas p.Val464Leu was only slightly reduced (66 ± 20% of WT levels; ns). Thus, rabphilin binding to 14‐3‐3 eta is significantly impaired in the p.Arg269Gln variant.

To assay interaction with Rab3a and SNAP25, we co‐expressed these constructs together with rabphilin 3a‐YFP in HEK cells and defined their cellular localization via immunofluorescence (Supporting Information Method Section). Rabphilin 3a WT was diffusely distributed when expressed alone but redistributed with Rab3a into vesicular compartments when they were coexpressed. A similar redistribution was observed with rabphilin 3a p.Arg269Gln and p.Val464Leu, indicating that they also interact with rab3a. In addition, both rabphilin 3a WT and p.Val464Leu recruited SNAP25 to these compartments (Figure 2d). Thus, the Val464Leu substitution in the C2A domain does not significantly disrupt binding of rab3a to the amino‐terminus, or SNAP25 to the adjacent C2B domain.

## 5 DISCUSSION

The condition described in this report expands the list of presynaptic forms of CMS recently identified with the assistance of WES. In comparison with postsynaptic forms, the newly discovered presynaptic CMS are rarer and often involve additional neurologic manifestations, including developmental delay, seizures, tremors and ataxia, which are not usually seen in postsynaptic CMS (Engel et al., 2016; Shen et al., 2014). At the electrophysiological level most of these presynaptic conditions show findings consistent with the Lambert Eaton Myasthenic Syndrome (LEMS). Moreover, in the few cases studied with intracellular microelectrodes, an impairment of the quantal release of neurotransmitter was confirmed as the primary mechanism of failure of neuromuscular transmission (Engel et al., 2016; Shen et al., 2014).

While the increment of CMAP areas in response to repetitive nerve stimulation at fast rates in our patient suggested a LEMS‐like condition, the characteristic decrement of CMAP amplitudes with slow rates of stimulation was not present, probably because the defect of transmission was mild and only detectable by single fiber EMG. In this regard, the mild neuromuscular deficit of our patient is in keeping with the modest phenotype that results from deletion of *RPH3A* in mice and *Caenorhabditis elegans* (Schlüter et al., 1999; Staunton et al., 2001).

In terms of the underlying pathogenic mechanism, it is unlikely that the reported syndrome is due to the *DOK7* 1124_1127dupTGCC mutation paired with an unidentified mutation in the same gene missed by direct sequencing and by the *DOK7* specific high density gene‐centric array analysis. Moreover, the phenotype linked to *DOK7* mutations is one of limb‐girdle myasthenia (Palace et al., 2007), not obvious in our patient; and does not associate with central nervous system symptoms, present in our patient. We cannot discount the possibility, however, that the heterozygous *DOK7* 1124_1127dupTGCC mutation acts as a genetic modifier that enhances the phenotype of the rabphilin mutations.

On the other hand, rabphilin 3a mutations are likely causative since they could impair neuromuscular transmission by disrupting SV release and/or recycling. As rabphilin 3a limits SV release (Burns et al., 1998; Deák et al., 2006), exocytosis could be less restrained by rabphilin 3a variants, ultimately leading to a depletion of SVs and failure in transmission. Alternatively, rabphilin 3a variants could perturb SV recycling, which is essential to maintain the vesicle pool. Indeed, injection of rabphilin 3a fragments at the squid giant synapse both inhibited transmitter release and reduced SV number by disrupting vesicle recycling (Burns et al., 1998). This appears to involve multiple rabphilin 3a domains and protein interactions as an NH2‐terminal rabphilin 3a fragment impaired endocytosis from the plasma membrane, while a C2A/B domain fragment disrupted a later step in vesicle trafficking.

Our analysis revealed defects in the motor nerve terminals of the patient consistent with this interpretation. In addition to a marked reduction in the density of SVs, we also observed DMBS, aberrant endosomes and degenerative lamellar bodies, which all suggest altered vesicular trafficking. Consequently, we propose that the rabphilin 3a variants impair one or more steps in SV recycling, such as endocytosis or endosomal processing, and thereby reduce the available vesicle pool and the efficiency of transmission. Potentially, this could occur by disrupting mechanisms that regulate rabphilin 3a function, as Arg269Gln lies adjacent to an activity‐dependent phosphorylation site (S272) that regulates 14‐3‐3 binding (Foletti & Scheller, 2001; Sun et al., 2003), and Val464Leu lies in the C2A domain that is regulated by Ca^2+^.

Also of interest are the similarities between rabphilin 3a and synaptotagmin 2, which is another molecule that contains two C2 Ca^2+^/phospholipid binding domains. Synaptotagmin 2 can also have positive and negative effects on SV release (Kochubey, Lou, & Schneggenburger, 2011), and interacts directly with proteins associated with the SNARE complex (Chapman, Hanson, An, & Jahn, 1995). Moreover, two different mutations in the C2B domain of synaptotagmin 2 result in autosomal‐dominant CMS, with disrupted SV exocytosis (Herrmann et al., 2014). It is possible, therefore, that single allele mutations in *RPH3A* may also result in disease. In fact, in our patient, we demonstrate a clear pathogenic effect for the Arg269Gln variant, whereas the effect of the Val464Leu variant remains unknown. Thus, we cannot discount the possibility monoallelic *RPH3A* variants causing autosomal‐dominant CMS, but with variable penetrance due to modifier effects from other variants in the same or other genes.

In summary, we report a patient with a complex phenotype characterized by failure of neuromuscular transmission, tremors and learning disabilities whose motor nerve terminals showed signs of disruptive SV homeostasis. The condition is attributed to sequence variants in *RPH3A* that alter both exocytosis and downstream endocytosis and SV recycling. The condition shows a favorable response to treatment with albuterol sulfate and encourages further research on the role of rabphilin 3a in human neurologic disorders of synaptic transmission.

## CONFLICT OF INTEREST

None.
