# Supplementary material for: Presynaptic congenital myasthenic syndrome with altered synaptic vesicle homeostasis linked to compound heterozygous sequence variants in RPH3A
Source: Mol Genet Genomic Med. 2018 Feb 14;6(3):434–40. doi: 10.1002/mgg3.370 (PMC6014458; doi:10.1002/mgg3.370)
Supplement: Supplementary file 1 [file MGG3-6-434-s001.pdf]

SUPPLEMENTARY DATA

Video S1

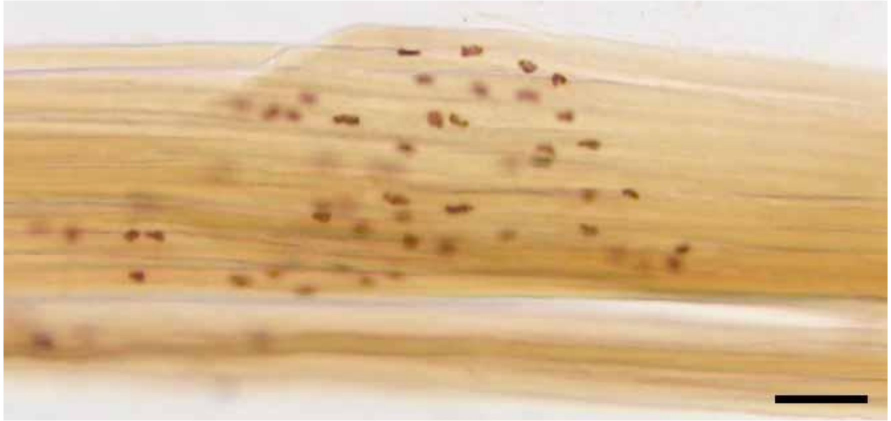

Figure S1. Acetylcholinesterase staining. A low magnification photograph of teased muscle bundles of the anconeus muscle showing normal expression of acetylcholinesterase and normal size and morphology of endplates (calibration mark = 100 µm).

Table S1 Morphometric data

	Patient	Control
EI*	10.44 ± 0.54 *(n=11)*	11.99 ± 2.46 *(n=11)*
Primary Synaptic Cleft (nm)	70.99 ± 2.82 *(n=12)*	74.0 ± 3.65 *(n=12)*
Presynaptic Area (µm^2)	7.41 ± 0.51 *(n=10)*	6.39 ± 1.22 *(n=11)*
Number of synaptic vesicles/µm^2	11.64 ± 1.65 *(n=10)* **	38.49 ± 4.95 *(n=11)*
Diameter of Synaptic Vesicles (nm)	47.22 ± 1.43 *(n=17)*	42.81 ± 2.48 *(n=11)*

* EI, endplate index (postsynaptic membrane length/presynaptic membrane length).

** (p<0.001)

Values reported as mean (SEM).

Method Section:

1. Whole Exome Sequencing (WES): The whole exome sequencing was performed in the DNA of the affected patient and her both parents by the commercial laboratory Gene Dx. The Agilent SureSelect XT2 All Exon V4 kit was used to target the exon regions of the genomes. These targeted regions were sequenced using the Illumina HiSeq 2000 sequencing system with 100bp paired-end reads. The DNA sequence was mapped to an analyzed in comparison with the published human genome build UCSC hg19 reference sequence. The targeted coding exons and splice junctions of the known protein-coding RefSeq gene were assessed for the average depth of coverage and data quality threshold values (Mean Depth of Coverage: 132x, Quality threshold: 98.3%). The XomeAnalyzer was used to evaluate sequence changes in the individual compared to other sequenced family members. All reported sequence variants in the proband and parental samples were confirmed by conventional di-deoxyDNA sequence analysis or other appropriate method. The mean depth of coverage refers to the sequence mean read depth across the XomeDx targeted region, defined as coding exons and splice junctions of Agilent SureSelect XT2. All Exon V4 kit targeted protein coding RefSeq genes. The quality threshold refers to the percentage of the XomeDx defined target region where read depth was at least 10x.coverage to permit high quality exome variant base calling and annotation.
Analysis: WES data for the patient and her parents was analyzed with the GeneDx's XomeAnalyzer (a variant annotation, filtering, and viewing interface for WES data), which includes nucleotide and amino acid annotations, population frequencies (from the NHLBI Exome Sequencing Project Exome Variant Server and 1000 Genomes), in silico prediction tools, amino acid conservation scores, and mutation references. Variants were filtered on the basis of inheritance patterns, lists of genes of interest, and phenotype and population frequencies, as appropriate. Synonymous variants and common variants (<0.01 in 1000Genomes< 0.01 in the GeneDx database) were ignored. Variants of low quality are also discarded for the analysis.

Table S2. Reported variants in genes with possible association with the patient's phenotype.

Gene	Disease	Mode of Inheritance	Variant	cDNA	Zygosity	Inherited from	Classification
ALG3	Congenital Disorder of Glycosylation	X-linked	E385G	C1154 A>G	Het	Mother	Unknown Significance
DSG2	Arrhythmogenic Right Ventricular Dysplasia 10	Autosomal Dominant	P269S	c.1885 C>T	Het	Mother	Unknown Significance
TMPO	Dilated Cardiomyopathy	Autosomal Dominant	P426L	C,1277 C>T	Het	Mother	Unknown Significance
DOK7	Congenital Myasthenic Syndrome	Autosomal Recessive	1124-1127dupTGCC		Het	Mother	Pathogenic
RPH3A	None Currently	Unknown	R269Q	c.806	Het	Father	Unknown

	Described			G>A			Significance
RPH3A	None Currently Described	Unknown	V464L	c.1390 G>T	Het	Mother	Unknown Significance

Table S3. Function of and phenotypes linked to genes with identified sequence variants.

Gene	Protein	Gene function and expression	Phenotype
ALG13	ALG13, UDP-N-acetylglucosaminyl-transferase subunit	The protein encoded by this gene is a subunit of a bipartite UDP-N-acetylglucosamine transferase that catalyzes the second sugar addition of the conserved oligosaccharide precursor in endoplasmic reticulum N-linked glycosylation. It is highly expressed in lymph nodes, skin and testis.	Epileptic Encephalopathy, Early Infantile
DSG2	Desmoglein-2	Desmogleins are calcium-binding transmembrane glycoprotein components of desmosomes, cell-cell junctions between epithelial, myocardial, and other cell types. They are highly expressed in colon.	Arrhythmogenic Right Ventricular Dysplasia 10
TMPO	Thymopoietin	The protein encoded by this gene resides in the nucleus and may play a role in the assembly of the nuclear lamina, and thus help maintain the structural organization of the nuclear envelope. It is highly expressed in lymph nodes.	Cardiomyopathy, dilated, 1A
DOK7	Dok7	The protein encoded by this gene is essential for neuromuscular synaptogenesis and plays a major role in the aggregation of acetylcholine receptors through the phosphorylation of the muscle specific kinase (MuSK).	Autosomal recessive limb-girdle congenital myasthenic syndrome type 1B.
RPH3A	Rabphilin 3A	The protein encoded by this gene is thought to be an effector for RAB3A, which is a small G protein that acts in the late stages of neurotransmitter exocytosis. The encoded protein may be involved in	Herein reported tremor, ataxia and muscle fatigability.

		neurotransmitter release and synaptic vesicle traffic. This gene is only highly expressed in the brain.	

2. *DOK7*-specific high density deletion/duplication analysis via gene-centric array CGH: This study was performed by the commercial laboratory Prevention Genetics using genomic DNA from the patient and a sex-matched reference sample labeled and hybridized on a microarray containing probes across the entire *DOK7* gene. The study was negative for deletion/duplication. The results are summarized in Figure S2.

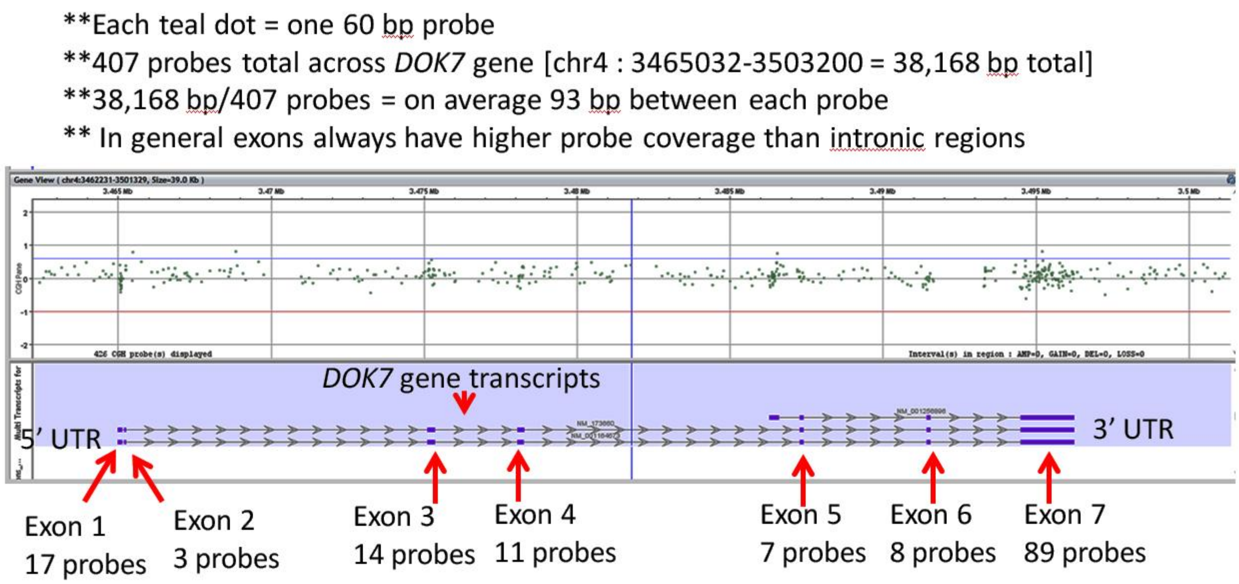

Figure S2. *DOK7* gene-centric array CGH. Summary of methods and results of the gene-centric array analysis.*

* Figure kindly provided by Dr. Angela Gruber from Prevention Genetics.

3. Pulldown Assay:

GST-14-3-3 fusion protein was produced in BL21 bacteria using a pGEX-2TK-14-3-3 eta GST vector (gift of Michael Yaffe; Addgene plasmid #13277) and purified on Glutathione 4B beads. Rabphilin 3a (WT, p.Arg269Gln and psVal464Leu) were introduced into x vector and expressed in HEK cells by transient transfection using Lipofectamine 3000 (Invitrogen). After 2 days expression, the cells were extracted in buffer containing 0.5% Triton X-100, 25 mM Tris, 25 mM glycine, 150 mM NaCl, 5mM EDTA, Halt protease inhibitor (ThermoScientfic) and phosphatase inhibitor cocktail (Calbiochem), and insoluble proteins pelleted by centrifugation. The soluble lysates were incubated with GST-14-3-3 eta Glut4B beads ~ 1 hr., and then washed three times in lysis buffer. Bound protein was eluted by boiling in 2x SDS-loading buffer, run on 8%

polyacrylamide gels, and immunoblotted with anti-rabphilin 3a antibody (sc-14687; Santa Cruz Biotech.) and IR800 anti-goat secondary antibody (LI-COR). Signal was visualized in an Odyssey scanner and quantified with ImageStudio software.

3. Immunostaining:

Rabphilin 3a–YFP (WT, p.Arg269Gln or p.Val464Leu), Rab3a and SNAP25 were expressed in HEK cells by transient transfection using Lipofectamine 3000 (Invitrogen). After 2 days expression, the cells were fixed in 2% paraformaldehyde in PBS, permeabilized with 0.5% Triton X-100, and immunostained with anti-Rab3a (sc-26552) or anti-SNAP25 antibody (sc-7538; Santa Cruz Biotech.) and AF594 anti-goat secondary antibody.
